# MicroRNA molecular profiling from matched tumor and bio-fluids in bladder cancer

**DOI:** 10.1186/s12943-015-0466-2

**Published:** 2015-11-14

**Authors:** David A. Armstrong, Benjamin B. Green, John D. Seigne, Alan R. Schned, Carmen J. Marsit

**Affiliations:** Departments of Pharmacology and Toxicology, Geisel School of Medicine at Dartmouth, Hanover, NH USA; Department of Surgery (Urology), Dartmouth-Hitchcock Medical Center, Lebanon, NH USA; Department of Pathology, Dartmouth-Hitchcock Medical Center, Lebanon, NH USA; Department of Epidemiology, Geisel School of Medicine at Dartmouth, Hanover, NH USA

**Keywords:** Bladder Cancer, MicroRNA, Urine, Plasma, Exosomes, NanoString, Droplet digital PCR

## Abstract

**Background:**

MicroRNAs have been identified as potential cancer biomarkers due to their presence and stability in many body fluids including urine and plasma, but the relationship of the pattern of expression of these messengers across various biological media has not been addressed and could provide important information in order to translate these biomarkers for epidemiologic or clinical use.

**Methods:**

We analyzed microRNA of matched FFPE-tumor tissue, plasma, urine exosomes (*n* = 16) and WBCs (*n* = 11) from patients with bladder cancer, using Nanostring miRNA assays and droplet digital PCR for validation. Pearson correlations were used to compare expression between media.

**Results:**

Numerous microRNAs were detected and overlapping from specific bio-specimen sources. MiR-4454 and miR-21 overexpression was found in three sources: tumor, WBCs and urine. Additionally, miR-15b-5p, miR-126-3p, miR-93-5p, and miR-150-5p were common to tumor/WBCs, while miR-720/3007a, miR-205, miR-200c-3p and miR-29b-3p common to tumor/urine. Significant associations were noted between the log-adjusted average miRNA counts in tumor vs. WBCs (*r* = 0.418 *p* < 0.001), and tumor vs. urine (*r* = 0.38 *p* < 0.001). No association was seen tumor vs. plasma exosome miRs (*r* = 0.07 *p* = 0.06).

**Conclusions:**

MicroRNA profiling from matched samples in patients shows a significant number of microRNAs up regulated in bladder tumors are identifiable in urine exosomes and WBCs of the same patient, but not in blood plasma. This study demonstrated varying relationships between miRNA detected in biological media from the same patient, and serves to inform the potential of urine-based microRNAs as biomarkers for bladder cancer and potentially other malignancies.

**Electronic supplementary material:**

The online version of this article (doi:10.1186/s12943-015-0466-2) contains supplementary material, which is available to authorized users.

## Background

Bladder cancer is the ninth most common cause of cancer worldwide with an estimated 429,000 new cases and 165,000 deaths in 2012 [1]. Bladder cancer is also one of the most expensive cancers to treat because of the high rate of local recurrence and the requirement for frequent long term follow-up with invasive and uncomfortable cystoscopic evaluations. The potential to replace cystoscopy with a reliable non-invasive evaluation of the urine or blood in the diagnosis and follow up of patients with bladder cancer is one of the “holy grails” of urologic oncology. Historically urologists have used urine cytology because of its high specificity, however the poor sensitivity, especially for patients with low grade tumors significantly limits the utility of this test. In recent years the FDA has approved a number of urine based tests that are variably used in clinical practice including Urovysion, which detects chromosomal abnormalities, BTA STAT and TRAK, which use monoclonal antibodies to detect complement factor H-protein, NMP22, which utilizes ELISA detection of a nuclear matrix protein in urine and and ImmunoCyt, which detects a mucin glycoprotein and carcinoembryonic antigen, both found on bladder tumor cells[2]. Although these provide some improvement over cytology there is a disparity of sensitivity and specificity especially across different cancer grades thus clinical utility remains sub-optimal. There is a clear unmet need for additional, improved urine (or other bio-specimen) based alternatives to cystoscopy for the screening, initial evaluation and follow up of bladder cancer [2].

MicroRNAs are a class of non-coding RNAs of 19 to 24 nucleotides that regulate gene expression through post-transcriptional, RNA interference, gene silencing pathways. The number of annotated human microRNA loci currently numbers at more than 1800 in the latest version of miRBase (v.21) [3]. Initially thought to act primarily intracellularly, circulating microRNAs have gained attention as extracellular messengers [4] Due to their inherent stability in bio-fluids these ribonucleotides have been looked at as potential non-invasive biomarkers to identify and monitor a variety of diseases. A number of studies across a variety of pathologies including pancreatic cancer [5] and colorectal cancer [6] are using panels of miRNAs as an approach to discovering new bio-fluid-based biomarkers. MicroRNA is an attractive candidate as a potential diagnostic biomarker not only due to high level of stability in body fluids but also its ability to be quantified on multiple platforms, including high-throughput efforts. The development of a non-invasive urine or blood-based miRNA or small RNA panel, either on its own or in conjunction with other available tests for improved detection of early stage bladder cancer could potentially improve the overall management of this disease by increasing the accuracy and decreasing the morbidity linked with current diagnostic approaches. Global expression patterns of miRNAs provides key opportunities with important practical applications; wherein a single miRNA or a signature of multiple miRNAs may improve risk stratification of patients and may supplement the histological diagnosis of urological tumors, particularly for bladder cancer [7].

In order for these miRNA to be applied diagnostically or prognostically, though, it is important to understand how their presence in various bio-fluids is related and is potentially representative of the patterns observed in the tumors themselves. In this study, we assessed the association between miRNAs expressed in tumor tissue of bladder cancer and matched bio-fluids. We used the NanoString nCounter microRNA assay to profile and compare each bio-specimen source for microRNA content and validated microRNA abundance by droplet digital PCR with TaqMan assays for specific microRNAs. These findings will lend themselves to an expanded validation study to assess the potential of urine exosome or white blood cell-derived microRNAs as diagnostic biomarkers in NMIBC.

## Results

### Characteristics of study participants

Demographics and characteristics of patients participating in this discovery study are presented in Table 1. The mean age of the participants was 69.38 ± 9.28 with 14 males and 2 females. The patients represented the clinical spectrum of bladder cancer from those with low grade non invasive tumors to high grade locally advanced muscle invasive tumors.

**Table 1:** Baseline clinical and pathological features of patients with bladder cancer

Gender	Age	Tumor grade
M	84	Low-grade Ta
M	63	Low-grade Ta
M	77	Low-grade Ta
M	66	Low-grade Ta
M	66	Tis
F	87	High-grade Ta
M	69	High-grade Ta
M	74	High-grade Ta
M	64	High-grade Ta
M	64	High-grade T1
M	72	High-grade T1 Tis
M	66	High-grade T1, Tis
M	79	High-grade T1, Tis
M	57	High-grade T2, Tis
F	52	High-grade T3
M	70	High-grade T4

### Comparison of microRNA profiles across bio-specimens

**Fig. 1:**
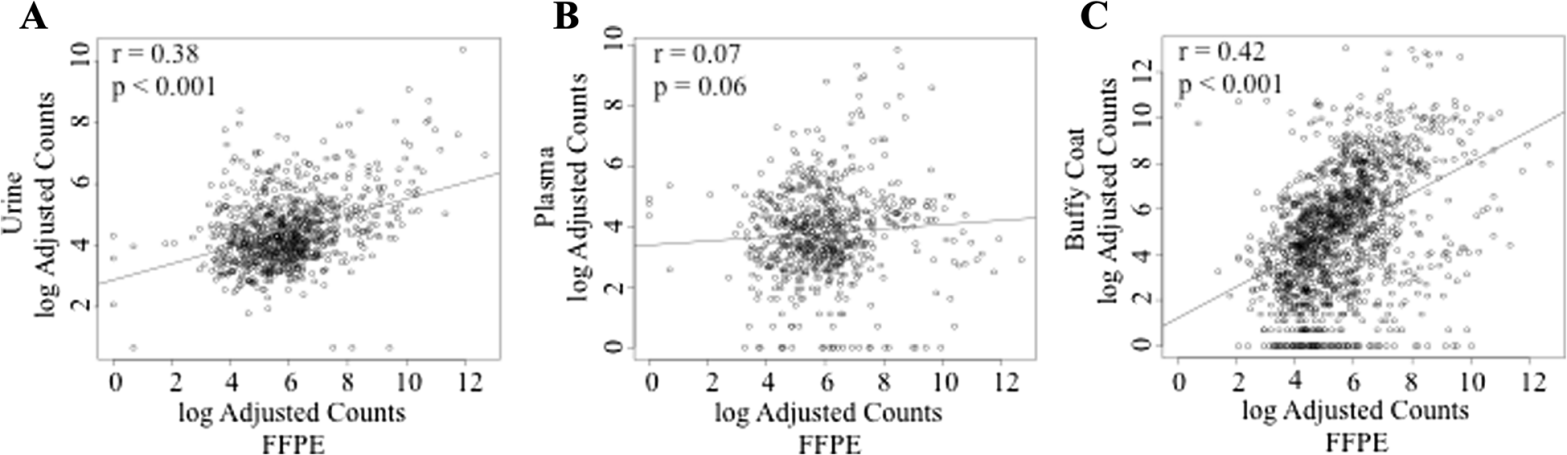
Association of FFPE-derived miRNA with bio-specimen-derived miRNA. MicroRNA abundance measured by NanoString miR assays in FFPE tumor tissue was compared to microRNA abundance in each additional bio-specimen. **a** urine exosomes (*n* = 16)(y-axis) and FFPE (*n* = 16) (x-axis). **b** plasma (*n* = 16) (y-axis) and FFPE (*n* = 16) (x-axis). **c** enriched-buffy coat (*n* = 11) (y-axis) and FFPE (*n* = 16) (x-axis)

The correlations between miRNA abundance in tumor vs. each of the other bio-specimens are presented in Fig. 1 as the normalized log adjusted NanoString counts per bio-specimen over the normalized log adjusted counts per tumor sample. The global correlations were moderate between tumor and urine exosomes (*r* = 0.38 *p* < 0.001) and between tumor and enriched-white blood cells fraction (WBCs) (*r* = 0.42 *p* < 0.001), however weak to no global correlation of tumor to cell-free plasma exosomes was observed (*r* = 0.07 *p* = 0.06).

**Fig. 2:**
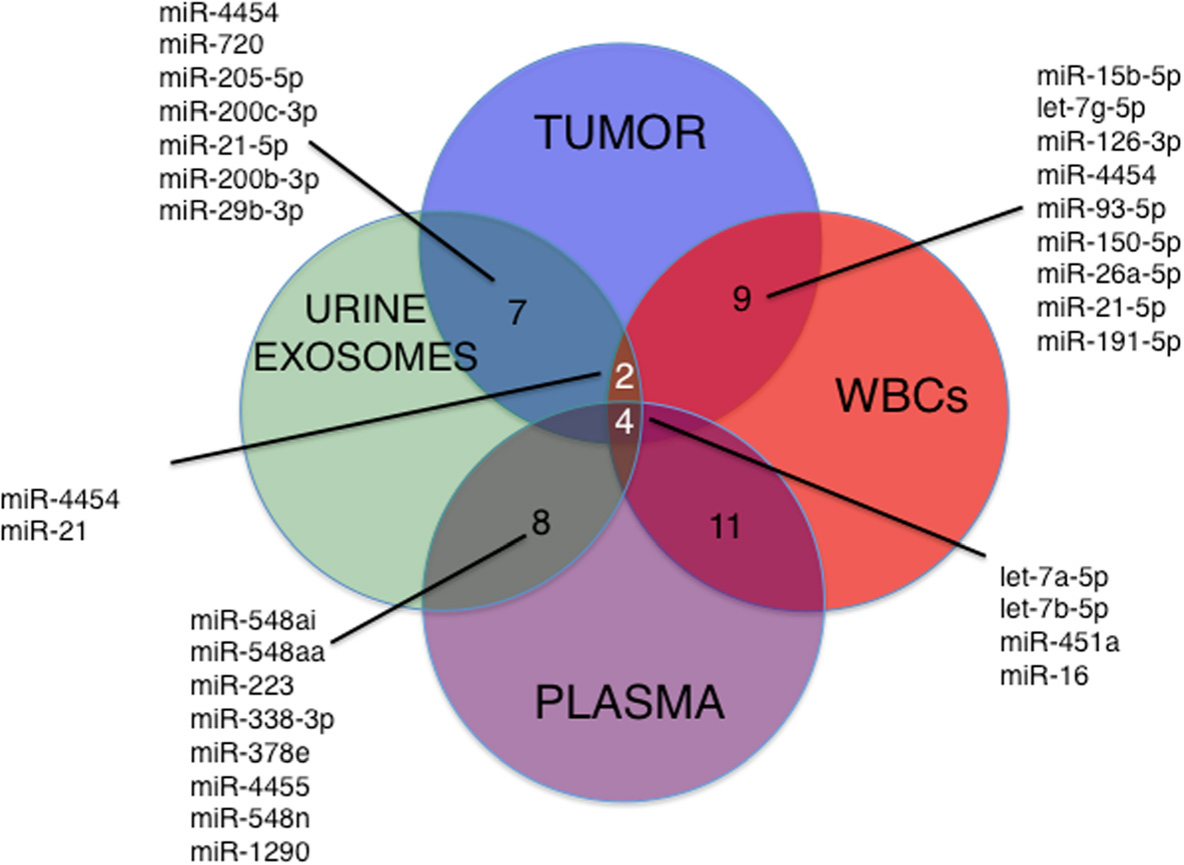
MicroRNAs common among bio-specimen sources in NMIBC. Overlapping distribution of microRNAs between the bio-specimens profiled by NanoString nCounter microRNA assay. Nine miRNAs are common between tumor and enriched-WBCs, seven miRNAs are common between tumor and urine exosomes, and two miRNAs are common between the three bio-specimens miR-4454 and miR-21

The top twenty-five miRNAs in highest abundance (in descending order) in each of the bio-specimen sources is shown in Table 2. The overlapping miRNAs identified between and amongst the bio-specimens sources is presented as a Venn diagram (Fig. 2). Two miRNAs were common amongst all bio-specimens – miR-4454 and miR-21. Six miRNAs and one transfer RNA fragment (tRF) were common to tumor and urine exosomes: miR-4454, miR-205-5p, miR-200c-3p, miR-200b-3p, miR-21-5p, miR-29b-3p and miR-720 /3007a - a tRF. Nine miRNAs were common between tumor and WBCs : miR-15b-5p, let-7g-5p, miR-126-3p, miR-4454, miR-93-5p, miR-150-5p, miR-26a-5p, miR-21-5p, and miR-191-5p.

**Table 2:** Top 25 MicroRNAs up-regulated in NMIBC

FFPE tumor-derived	Urine: cell-free and exosome-derived	Plasma: circulating and exosome-derived	WBC-derived (*n* = 11)
miR-4454	miR-4454	miR-451a	miR-451a
miR-720	miR-548ai	miR-16-5p	miR-16-5p
let-7a-5p	miR-320e	miR-223-3p	miR-144-3p
miR-205-5p	miR-451a	miR-548ai	let-7a-5p
miR-145-5p	miR-548aa	miR-548aa	miR-15b-5p
miR-200c-3p	miR-200c-3p	miR-378e	miR-223-3p
let-7b-5p	miR-720	miR-338-3p	let-7b-5p
miR-21-5p	miR-21-5p	miR-4455	let-7g-5p
let-7g-5p	miR-4516	miR-126-3p	miR-142-3p
miR-125b-5p	miR-223-3p	has-miR-23a-3p	miR-126-3p
miR-451a	miR-338-3p	miR-106a-5p + miR-17-5p	miR-4454
miR-23a-3p	miR-16-5p	miR-25-3p	miR-15a-5p
miR-200b-3p	miR-200b-3p	miR-92a-3p	miR-106a-5p + miR-17-5p
miR-29b-3p	miR-4455	miR-142-3p	miR-150-5p
miR-143-3p	miR-378e	miR-20a-5p + has-miR-20b-5p	miR-93-5p
miR-16-5p	miR-548n	miR-144-3p	miR-20a-5p + has-miR-20b-5p
miR-126-3p	miR-205-5p	miR-19b-3p	miR-26b-5p
miR-26a-5p	miR-29b-3p	let-7a-5p	miR-26a-5p
miR-191-5p	miR-1290	let-7b-5p	miR-25-3p
miR-15b-5p	miR-22-3p	miR-1290	miR-21-5p
miR-150-5p	let-7b-5p	miR-26a-5p	miR-106b-5p
miR-1260a	miR-1972	miR-302b-3p	let-7f-5p
miR-4286	miR-30a-5p	miR-22-3p	miR-191-5p
miR-23b-3p	miR-23a-3p	let-7g-5p	let-7i-5p
miR-93-5p	let-7a-5p	miR-93-5p	miR-19b-3p

As well as analyzing the entire patient cohort as a pool, we have compared the microRNA abundance directly in matched tumor and urine exosome bio-specimens. With the exception of miR-21-3p (in 4 Ta samples) all of the seven overlapping miRs presented in Fig. 2 are detectable from both tumor and urine exosomes in matched samples (16/16). Additionally, all of the nine overlapping miRs presented in Fig. 2 are detectable from directly matched tumor and WBC samples (16/16).

As this profiling may be most useful in non-invasive evaluation of low grade tumors, we examined specifically the relationships between miRNA abundance in matched bio-specimens from Ta patients only, with some limitations in the number of available matched samples (urine *n* = 4 or WBCs *n* = 3). In these Ta patients, comparing tumor vs. urine exosomes, miR-205-5p and miR-21-5p drop out of the Top 50 in abundance leaving miR-4454, 720, 200c-3p, 29b-3p and 200b-3p as potential candidates for further investigation in urine exosomes. Comparing miR abundance in tumor vs. WBCs, miR-21-5p and miR-191-5p drop out of the Top 50 in abundance, leaving miR-126-3p, 93-5p, let-7g-5p, 26a-5p, 15b-5p, 150-5p and 4454 as potential candidates for follow-up investigation in Stage Ta BCa WBCs.

**Fig. 3:**
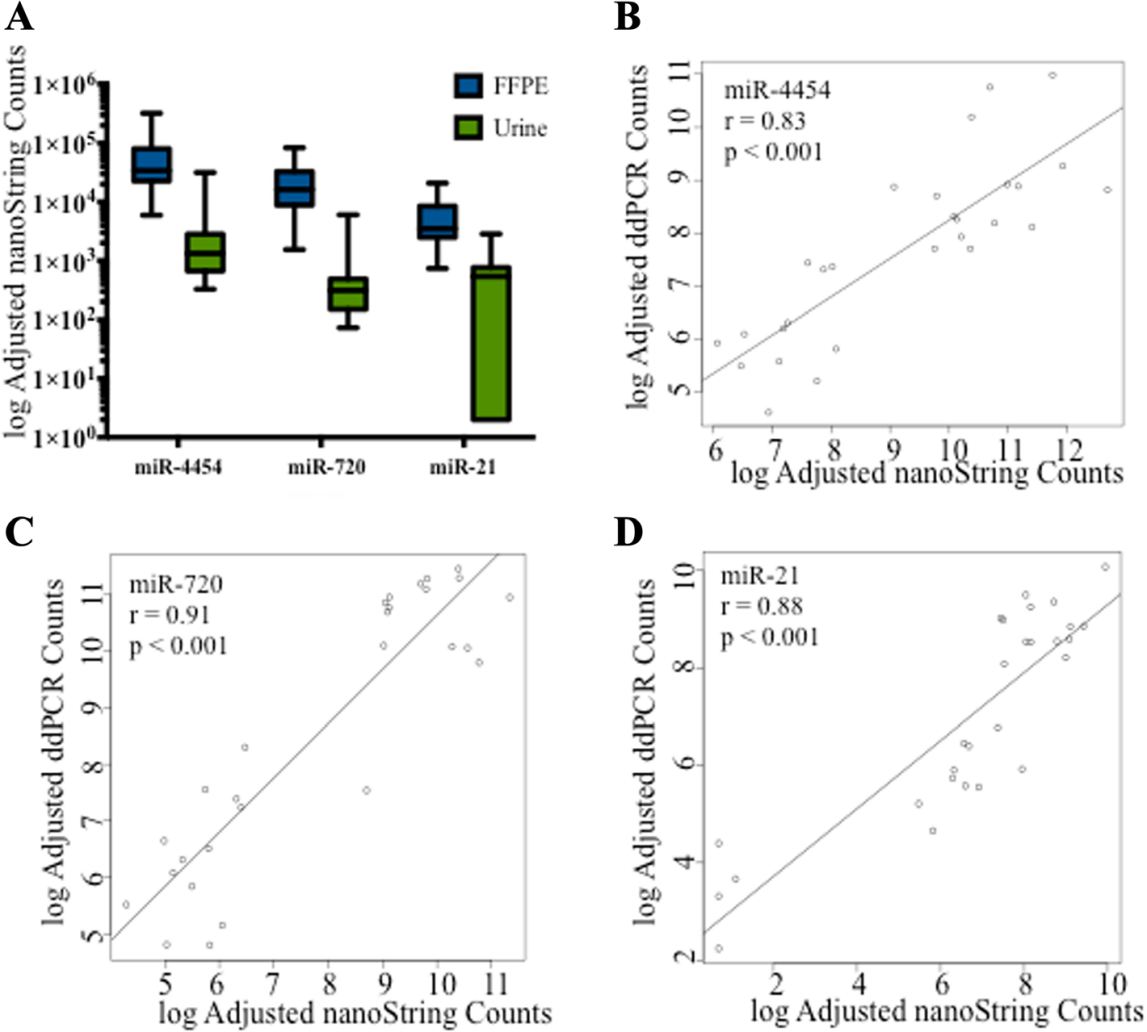
NanoString Counts Comparing FFPE-tumor with Urine Exosomes and Correlation of NanoString Counts to ddPCR Counts. NanoString nCounter miRNA assays were used to profile microRNAs in tumor and cell-free urine and subsequently compared in three of the highest abundance miRNAs (miR-4454, miR-720, and miR-21) (**a**). ddPCR/ Taqman assays were used for independent validation of specific microRNAs. Correlations were high between NanoString and ddPCR for miR-4454 (**b**), miR-720 (**c**) and miR-21 (**d**)

Next we focused on several of the miRNAs of highest abundance in both tumor and urine exosomes to obtain an independent quantitative measurement and validation of the Nanostring miRNA assay results. NanoString miRNA assay counts for miR-4454, miR-720/3007a and miR-21 for both FFPE-tumor and urine exosomes are presented in Fig. 3a. Correlations between NanoString counts and and independent ddPCR miRNA assays were strong: miR-4454 (*r* = 0.83 *p* < 0.001) (Fig. 3b), miR-720/3007a (*r* = 0.91 *p* < 0.001) (Fig. 3c) and miR-21 (*r* = 0.88 *p*, 0.001) (Fig. 3d).

### miR-4454 and miR-720/3007a are not correlated to hemolysis marker miR-451a in urine exosomes

**Fig. 4:**
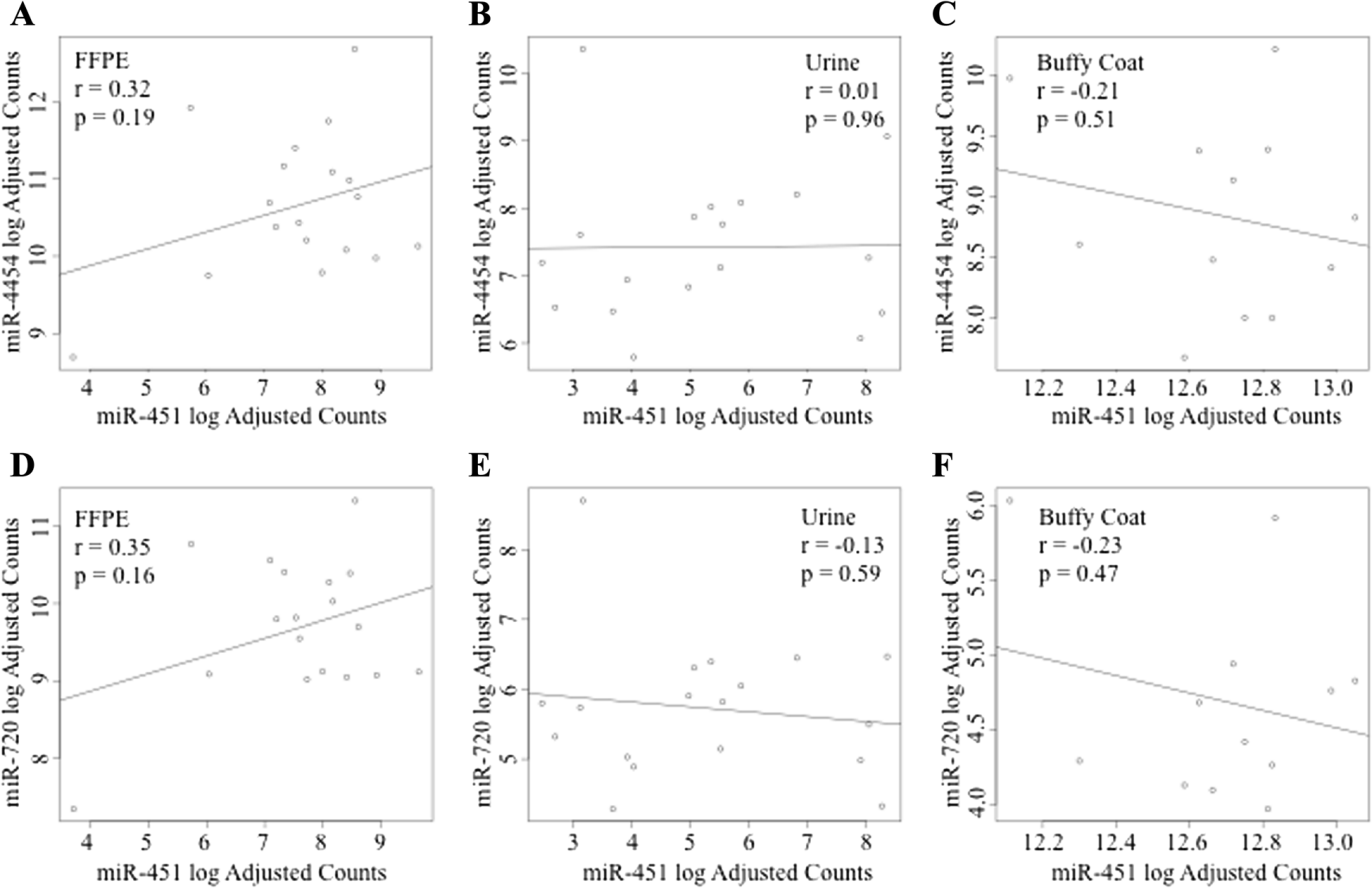
Correlation of miR-451a versus miR-4454 & miR-720/3007a in FFPE, urine exosomes and enriched-buffy coat. NanoString nCounter microRNA assay was used to determine the abundance of miR-451a, miR-4454 and miR-720/3007a across patient samples in FFPE-derived RNA, urine exome miRs and enriched-WBCs RNA. Moderate correlations were seen in FFPE: miR-4454 vs miR-451a (**a**) (*r* = 0.32 *p* = 0.19), and miR-720 vs miR-451a (**d**) (*r* = 0.35 *p* = 0.16). Weak to no correlation seen in urine exosomes: miR-4454 vs miR-451a (**b**) (*r* = 0.01 *p* = 0.96) and miR-720 vs miR-451a (**e**) (*r* = 0.13 *p* = 0.59). Moderate negative correlation was seen in enriched-buffy coat : : miR-4454 vs miR-451a (**c**) (*r* = −0.21 *p* = 0.51) and miR-720 vs miR-451a (**f**) (*r* = − 0.23 *p* = 0.47)

Pritchard et al. [8] have shown miR-451a present in hemolyzed plasma. Using miR-451a as a surrogate measure of red blood cell content or hemolysis across tissue or bio-fluids, we examined if the other highly abundant miRNAs identified may be similarly representative, by examining their correlation with this marker. Moderate correlations were seen in FFPE : miR-4454 vs miR-451a (Fig. 4a) (*r* = 0.32 *p* = 0.19), and miR-720 vs miR-451a (Fig. 4d) (*r* = 0.35 *p* = 0.16). Weak to no correlation seen in urine exosomes : miR-4454 vs miR-451a (Fig. 4b) (*r* = 0.01 *p* = 0.96) and miR-720 vs miR-451a (Fig. 4e) (*r* = 0.13 *p* = 0.59). Moderate negative correlation was seen in enriched-WBCs : miR-4454 vs miR-451a (Fig. 4c) (*r* = −0.21 *p* = 0.51) and miR-720 vs miR-451a (Fig. 4f) (*r* = − 0.23 *p* = 0.47).

## Discussion and conclusions

The goal of this study was to examine the co-occurrence of miRNA profiles identifiable in tumor samples with those found in accessible other bio-specimen samples from the same individual, in order to demonstrate which bio-specimen may be the most useful for further examinations of clinical biomarkers. Previous discovery studies have commonly focused on only one bio-specimen source for microRNA analysis whether that be urine, plasma, or serum [9]. With urine as the bio-specimen source, studies to date have compared microRNA levels in bladder cancer vs. control using either total urine [10] or urine cell pellet [11]. One study however, has examined paired microRNA expression in tumor and total urine [12]. With plasma/serum as a source, several studies have analyzed bio-fluid microRNA content correlated with tumor stage [2, 13], while only one group has conducted a tumor/plasma paired analysis [14].

Our study is the first to report microRNA molecular profiling from four matched bio-specimen sources: tumor, urine exosomes, enriched-white blood cells and plasma exosomes. Our approach is unique in profiling bladder tumor tissue and multiple additional bio-specimens from the same individual.

Our initial strategy was to focus on profiling from FFPE-derived tumor and urine exosome miRNAs, as this would seem from the perspective of a validation study, the approach with the highest level of patient comfort and likelihood of success. MicroRNA profiling analysis from a tumor/urine exosome pairing revealed a group of six microRNAs and one transfer RNA fragment (tRF) with good correlation between the bio-specimen sources, two in particular, miR-4454, and miR-720/3007a have not previously been reported in bladder cancer.

Of the top 25 highest abundance miRNAs across the four bio-specimens, miR-4454 was expressed at high levels in three out of four bio-specimens. Limited studies have previously identified miR-4454 involvement or regulatory function in cancer or even in normal metabolic pathways. miR-4454 has been noted as a common component among three sub-populations of extracellular vesicles secreted in a human colon carcinoma cell line [15]. Additionally, miR-4454 has been pointed out as a possible target for TNF-α systemic inflammation via NFκB pathway regulation [16]. Target Scan (Release 6.2) [17] reports potential binding sites in the 3’-UTR for several target genes for miR-4454, including: ABCD1, CNKSR2, EGR3 and SPARC.

Elevated cellular levels of transfer RNA are a hallmark of proliferative diseases, such as cancer [18]. Our work here is the first to identify a transfer RNA fragment (tRF) – miR-720/3007a – in NMIBC tumors and in urine exosomes from those same patients. tRFs are 14–32 base long single-stranded RNA derived from mature or precursor tRNA and are distinct from the stress-induced tRNA fragments created by cleavage in the anti-codon loop. tRFs have been grouped into 3 classes (tRF-1, tRF-3, and tRF-5) as well as 5 subclasses depending upon their cleavage site within a mature tRNA [19]. Mechanistically, tRFs have been shown to be important for cell-cycle progression and for regulating the dynamics of RISC [19]. MiR-720, originally falsely annotated as a microRNA has been reclassified as a tRF [20]. This recently discovered class of small RNA has been found to be present in diverse organisms at read counts comparable to miRNAs. Currently, there is a debate about their biogenesis and function [19]. In a recent report, miR-720/3007a as a tRF, has been found in high abundance in extracellular vesicles shed from a breast cancer cell line [21].

There are more than a dozen reports where miR-720/3007a may have been misclassified as a microRNA rather than a tRF, but its physiological effects or functions may still be notable. miR-720 has been described in several cancer settings where a exact regulatory role appears to be dependent on the cancer type. Wang et al. [22] have suggested miR-720 as a promoting factor in the development of colorectal cancer, where its influence was noted in promoting cell growth, migration and invasion *in vitro*. TargetScan reports miR-720 3’-UTR binding sites for eight possible target genes, amongst which include: DNMT3A, ACVR1B, FOXG1, and FGF14. Clearly, universal recognition of miR-720/3007a under the tRF classification is important moving forward. Perhaps from the standpoint of urine-based diagnostic markers, tRFs may have as high a potential as miRNAs for creation of a panel of unique markers for any particular pathology.

The miR-200 family contains several of the miRNAs we have identified in the tumor/urine exosome association, which have previously been reported in bladder cancer studies [9].

The miR-200 family was first identified as a potential regulator of epithelial-to-mesenchymal transition (EMT) in bladder cancer cells and the different family members appear to control the EMT by targeting ZEB1, ZEB2, and EGFR [7]. Additionally, expression of family member miR-200c has been reported correlated with early stage bladder tumor progression with DNA promoter methylation as the regulatory mechanism [7].

miR-21 is one of the most highly expressed members of the small non-coding microRNA family in many mammalian cell types. Its expression is further enhanced in many disease states including solid tumors, cardiac injury, and inflamed tissue [23]. There are reports of miR-21 measured in urine in various pathological conditions [24], so it is not particularly surprising that we are able to detect it here in NMIBC. Nevertheless, miR-21 is a potential candidate for a miR panel due to its presence in all later stage bio-specimens we profiled.

The effects of hemolysis occurring during bio-fluid collection or processing can have considerable impact on the levels of certain microRNAs detected in hemolyzed samples [8, 25]. miR-451a is a well known microRNA released from red blood cells during hemolysis [25]. Using miR-451a as a surrogate measure of hemolysis and its potential influence on microRNA abundance across our bio-specimen sources, we examined the correlation of miR-451a versus two candidate miRNAs identified in the NanoString miR assay: miR-4454 & miR-720/3007a - in tumor, urine exosomes and enriched-WBCs. As expected due to the presence of RBCs and transfer RNA within tissue itself, we noted moderate correlation between miR-451a and miR-4454 and miR-720/3007a in the FFPE-derived RNA. No association was seen in urine exosome miR-451a vs miR-4454 or miR-720/3007a, enhancing the sensibility of urine exosomes as a potential source for specific diagnostic biomarkers.

A number of other recent studies have identified specific microRNAs as potential diagnostic/prognostic markers for bladder cancer including: Kim et al. (miR-214) [26], Hanke et al. (miR-126, miR-182, and miR- 199a)[10] and Ratert et al. (miR-20a, miR-106b, miR-130b, miR-141, miR-200a, miR-200a*, and miR-205) [27]. All of these microRNAs were detected across our bio-specimens but their abundance did not place them in the Top 25 in any bio-specimen within study (with the exception of miR-205 in FFPE-tumor), therefore we did not highlight them nor pursue their analysis further. This difference in detection may be related to differences in the populations studied, our small sample size, and differences in the methodologies of detection, as prior studies relied on real-time PCR or microarray approaches, compared to our use of the Nanostring system of direct molecule counting.

This study has a number of limitations. It is a descriptive study; more specific examinations of microRNAs or small RNAs individually or as a panel should be performed to examine utility as non-invasive or minimally-invasive diagnostic tools. Studies of larger sample size are also needed in order to assess clinical correlations with these small RNA. Also, we are aware of the challenges in clinical studies linked to sample collection, blood component fractionation, and clinically archived samples, related to red blood cell or platelet microRNA contamination due to lysis/hemolysis. A number of microRNAs have been identified associated with hemolysis including: miR-451a, miR-16, miR-486-5p and miR-92a [8], along with an additional group of microRNAs identified associated with platelets (miR-223, miR-16, miR-126 and miR-93 [28]). We do see some of these microRNAs in our bio-specimen profiles and would suggest they are not the ideal candidates to include in follow-up studies. Finally, the focus of this study was not to explore the potential mechanisms of miRNAs or small RNAs in NMIBC.

These limitations notwithstanding, our study has a number of strengths. This is the first to report microRNA molecular profiling from four matched bio-specimen sources: FFPE-derived bladder cancer tumor, urine: cell-free and exosome-derived, enriched-white blood cells and plasma: circulating and exosome-derived. We have identified six microRNAs that are common between tumor and urine exosomes, nine microRNAs that are common to tumor and an enriched-white blood cell fraction and two microRNAs that are common to all three tissue sources in NMIBC. Based on our findings, both urine exosome and enriched-white blood cell-derived microRNA profiling correlates well with bladder tumor microRNA abundance and may possess significant potential as sources of microRNA for diagnostic biomarker development in bladder cancer. Cell-free plasma exosome-derived and circulating microRNA, which does not correlate with bladder tumor microRNAs, probably is not a good choice for biomarker development in NMIBC. We believe that our approach of surveying as many bio-specimen sources as possible matched to the primary tumor is applicable not only to bladder cancer but could be applied in almost any area of human pathology investigation. Additionally, this the first study to report a detectable transfer RNA fragment species from tumor and matched urine exosomes.

This study presents a number of perspectives to consider related to methodology, reproducibility, bio-specimen selection and downstream biomarker development. For methodology the NanoString microRNA platform offers an opportunity to profile hundreds of microRNAs simultaneously with high sensitivity, precision and reproducibility, along with the ability to generate a direct molecule count without amplification. From the perspective of bio-specimen source, our selection of urine exosomes for analysis over total urine presents two advantages. Exosomes contain distinct cargo that closely mirrors the inner compartments of their cells of origin [29], and in the case of NMIBC we hope to take advantage of measuring miRs secreted or shed directly from the transitional epithelium of the bladder. Secondly, exosomes protect their internal microRNA contents from endogenous RNAses and lend long-term storage stability to the microRNA contained in cell-free urine. This allows for establishment of a standardized protocol moving forward, whereas total urine (containing cells) may be subject to cell lysis during multiple freeze/thaws, potentially altering measurable microRNA content from one analysis to the next.

The development of a non-invasive urine or blood-based miRNA/small RNA panel, either on its own or in conjunction with other available tests for improved detection of early stage bladder cancer could potentially improve the management of this disease. Future studies can now address if specific miRNAs or tRFs that we have identified in these bio-specimens may potentially serve as diagnostic biomarkers for NMIBC.

## Methods

### Bio-fluid collection and storage

Investigations were performed after approval by the Committee for the Protection of Human Subjects at Dartmouth College and in accordance with an assurance filed with and approved by the U.S. Department of Health and Human Services. Written informed consent was obtained from each subject. Subjects were identified and recruited into the study during their diagnostic appointment but prior to tumor resection. Urine and peripheral blood samples were collected during that visit or at a subsequent visit, prior to tumor resection. Additional details on processing and storage are provided in Supporting Methods section.

### Total RNA/MicroRNA extraction, purification and quantification

Total RNA and microRNA were extracted using assorted Norgen Purfication Kits per manufacturer’s instructions. Quantification of RNA was by Nanodrop 2000 and Agilent BioAnalyzer 2100 (see Additional file 1). BioAnalyzer electropherograms of total RNA or microRNA isolated from bio-specimens are shown in Additional file 2.

### NanoString microRNA assays and droplet digital PCR

NanoString nCounter microRNA assay and droplet digital PCR were performed according to manufactures’ instructions (see Additional file 1). Correlations of NanoString nCounter microRNA assay technical replicates are presented in Additional file 3.

### Statistical analysis

Pearson correlations were used in all analyses and data was analyzed using the R package version 3.1.0. Graphs were created within the R package or GraphPad Prism v 6.0. (see Supporting Methods for additional details).

## Additional files

Additional file 1:
**Methods (Expanded)** (DOCX 112 kb) Additional file 2: Bio-specimen Electropherograms. BioAnalyzer Electropherograms of Total RNA or microRNA isolated from Bladder Cancer Bio-specimens. Total RNA or microRNA was isolated from FFPE tumor slices, urine exosomes, enriched WBCs (Buffy) as described in methods. 1 ul per sample run on BioAnalyzer 2100 with the Small RNA Chip kit. MIcroRNA seen at 20–40 nt (37–45 s), additional peaks in electropheregram represent tRNA and other small RNAs. (TIF 1521 kb) Additional file 3: NanoString Technical Replicates. Correlations of NanoString nCounter microRNA assay technical replicates. Technical replicates of FFPE-derived total RNA (A) and urine exosome microRNA (B) were run on the NanoString nCounter microRNA assay. Strong correlation was seen in both the FFPE samples (*r* = 0.96 *p* <0.01) and the urine exosome samples (*r* = 0.95 *p* < 0.001). (TIF 1521 kb)

## Abbreviations

FFPE: Formalin-fixed paraffin-embedded
miR/miRNA: MicroRNA
NMIBC: Non-muscle invasive bladder cancer
PCR: Polymerase chain reaction
tRF: Transfer RNA fragment
WBC: White blood cells
