# Supplementary material for: MicroRNA molecular profiling from matched tumor and bio-fluids in bladder cancer
Source: Mol Cancer. 2015 Nov 14;14:194. doi: 10.1186/s12943-015-0466-2 (PMC4650939; doi:10.1186/s12943-015-0466-2)
Supplement: Additional file 1: — Methods (Expanded) (DOCX 112 kb) [file 12943_2015_466_MOESM1_ESM.docx]

Additional File 1

**Biofluid collection and storage**

Whole blood was collected in a Vacutainer EDTA(K2) plastic tube (Becton Dickinson, CA) and fractionated by centrifugation at 1,500 _×_ g for 20 minutes at 20°C. Plasma, enriched-WBCs, and red blood cells were aliquoted into cryogenic vials (Corning Incorporated, Corning, NY) and stored at -80C. Urine was collected mid-stream in Clikseal containers (Therapak Corp, Buford, GA), and subsequently transferred to 15 ml conical tubes (VWR, Radnor, PA) for centrifugation. Urine was centrifuged twice, first at 1,200 x rpm for 20 minutes at 20C. Supernatant was transferred to fresh conical tube and centrifuged at 2,500 x rpm for 20 minutes at 20 C. Final supernatant was aliquoted into cryogenic vials and stored at -80C.

**Total RNA / MicroRNA Extraction, Purification and Quantification**

Total RNA was extracted from 3x 20 μM slices of FFPE-tumor tissue using Norgen FFPE RNA/DNA Purification Plus Kit (Norgen Biotek Corp., Thorold, ON, Canada). Circulating and exosomal plasma microRNA was isolated from a 200 ul plasma volume using Norgen Plasma/Serum Circulating and Exosomal RNA Isolation Kit. Cell-free microRNA from urine was obtained using Norgen Urine Exosome RNA Isolation Kit from a 4 ml volume of cell-free urine. Enriched-WBC RNA was isolated using Norgen Total RNA Purification Kit. All protocols were performed according to manufacturer’s instructions. Plasma and urine microRNA was purified and concentrated using Amicon Ultra 0.5 columns (Millipore, Billerica,MA) (Erroll Reuckert, NanoString personal communication). Briefly, eluted microRNA was brought to a total volume of 420ul in nuclease-free water, column/collection tube loaded and centrifuged at 14,000 x g for 20 minutes at 20C. Column was placed in a fresh collection tube in inverted position and centrifuged 8,000 x g 2 minutes at 20C. Recovered miRNA was concentrated on Speed Vac (ThermoFisher Scientific, Waltham, MA) 15-20 minutes at 20 C. Total RNA was quantified using a Nanodrop 2000 (ThermoFisher Scientific). MicroRNA was quantified on the Agilent Bioanalyzer 2100 (Agilent Technologies, Santa Clara, CA) using the Small RNA Chip assay (Electropherogram profiles and miRNA concentrations are representatively shown in additional file 2).

**Nanostring microRNA Assays and Droplet digital PCR**

RNA samples were aliquoted and stored at -80 C and samples were thawed only once for expression analysis. The digital multiplexed NanoString nCounter human v2 microRNA expression assay (NanoString Technologies, Seattle,WA) was performed according to manufacturers instructions with total RNA or miRNAs extracted as above. Briefly, 3-10 ng microRNA (plasma/urine) or 100 ng total RNA (FFPE/WBCs) samples were prepared by ligating a specific miR-tag onto the 3’ end of each mature miR followed by an overnight hybridization (65 C) to nCounter Reporter and Capture probes. Excess Reporter and Capture probes are washed away using the automated nCounter sample prep station and probe/target complexes are aligned and immobilized in the nCounter Cartridge. Cartridges are then placed in the nCounter digital analyzer for data collection. nSolver Analysis software (NanoString) (V2.5) was used for data analysis including background correction by subtracting the mean of the six negative controls included on the NanoString platform and normalization using the average geometric mean of the top one hundred probes detected. Technical replicates showed strong correlation illustrated in additional file 3.

First strand reaction was performed with TaqMan microRNA reverse transcription kit (Life Technologies, Grand Island, NY) following manufacturer’s protocols. Droplet digital PCR (ddPCR) was performed with the QX100 Droplet Digital PCR system as follows: 20 μL of the reaction mixture containing 8 μL of cDNA solution, 10 μL of digital PCR^TM^ Supermix (Bio-Rad, Hercules, CA), and 1 μL of Taqman primer/probe mix (Life Technologies) and DEPC H_2_O was loaded into a plastic cartridge with 70 μL of QX100 Droplet Generation oil and then placed into the QX100 Droplet Generator. The droplets generated from each sample were transferred to a 96-well PCR plate (Eppendorf, Germany). PCR amplification was carried on a Veriti thermal cycler (Life Technologies) at 95°C for 10 min, followed by 40 cycles of 95°C for 30 seconds and 60°C for 1 min, then 1 cycle of 98°C for 10 min, ending at 4°C. The plate was then loaded on Droplet Reader for analysis. Absolute quantification of each miRNA was calculated from the number of positive counts per panel using the Poisson distribution, as previously described ([27](#_ENREF_27)). The quantification of the target miRNAs was presented as the number of copies/μL of PCR mixture.
