# Supplementary figures and images for: MicroRNA molecular profiling from matched tumor and bio-fluids in bladder cancer
Source: Mol Cancer. 2015 Nov 14;14:194. doi: 10.1186/s12943-015-0466-2 (PMC4650939; doi:10.1186/s12943-015-0466-2)

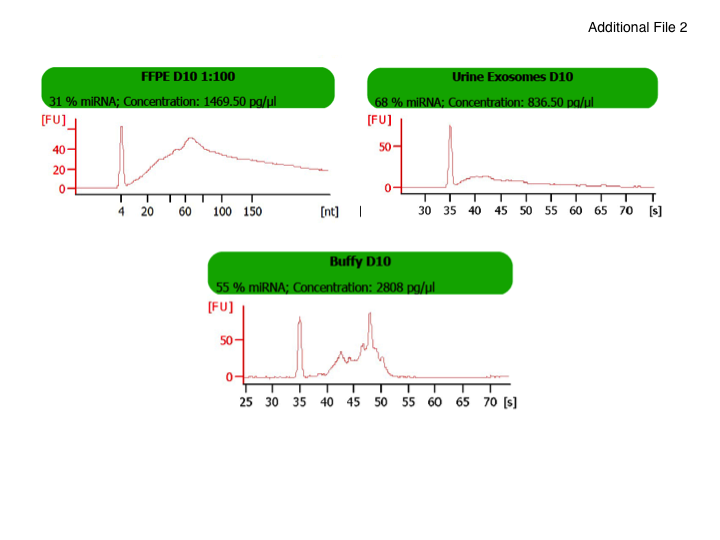

Supplement: Additional file 2: Bio-specimen Electropherograms. — BioAnalyzer Electropherograms of Total RNA or microRNA isolated from Bladder Cancer Bio-specimens. Total RNA or microRNA was isolated from FFPE tumor slices, urine exosomes, enriched WBCs (Buffy) as described in methods. 1 ul per sample run on BioAnalyzer 2100 with the Small RNA Chip kit. MIcroRNA seen at 20–40 nt (37–45 s), additional peaks in electropheregram represent tRNA and other small RNAs. (TIF 1521 kb) [file 12943_2015_466_MOESM2_ESM.tif]

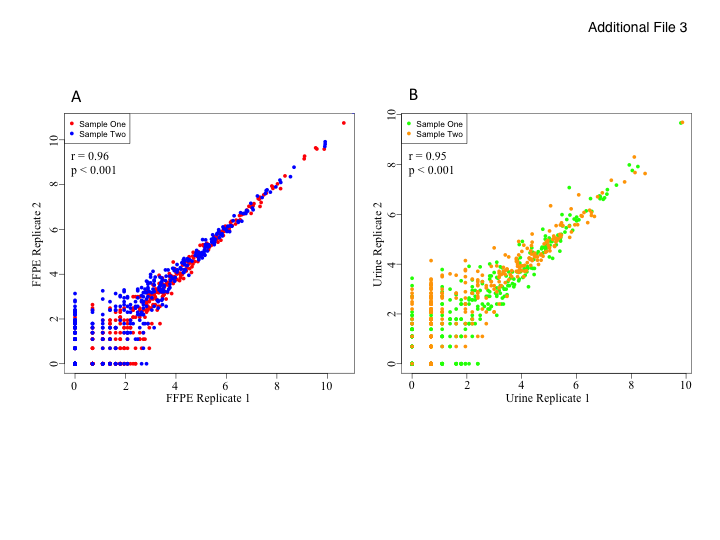

Supplement: Additional file 3: NanoString Technical Replicates. — Correlations of NanoString nCounter microRNA assay technical replicates. Technical replicates of FFPE-derived total RNA (A) and urine exosome microRNA (B) were run on the NanoString nCounter microRNA assay. Strong correlation was seen in both the FFPE samples (r = 0.96 p <0.01) and the urine exosome samples (r = 0.95 p < 0.001). (TIF 1521 kb) [file 12943_2015_466_MOESM3_ESM.tif]
